# Get Rid of German Control

**Published:** 1918-12

**Authors:** 


### Get Rid of German Control.

Investigations have revealed a clever attempt on the part of alien enemies to get control of the drug, chemical, and surgical business of the United States. The plans were well covered and would never have been discovered but for the war. Now that citizens of the United States have been shown the machinations of these Germans, care should be taken to see that in future no aliens shall get control of so important a branch of American manufacture and trade.

